# Balanced DNA-to-cytoplasm ratio at the 2-cell stage is critical for mouse preimplantation development

**DOI:** 10.1016/j.isci.2025.114416

**Published:** 2025-12-12

**Authors:** Tao Pan, Natsumi Taira, Miho Ohsugi

**Affiliations:** 1 Department of Life Sciences, Graduate School of Arts and Sciences, The University of Tokyo, 3-8-1 Komaba, Meguro-ku, Tokyo 153-8902, Japan; 2 Department of Biological Sciences, Graduate School of Science, The University of Tokyo, 7-3-1 Hongo, Bunkyo-ku, Tokyo 113-8654, Japan

**Keywords:** cell biology, developmental biology, genetics, molecular biology

## Abstract

Among vertebrates, mammalian haploid embryos are highly prone to early developmental arrest for reasons that remain unclear. Using mouse preimplantation embryos with altered cell size and ploidy, we show that arrest primarily results from an imbalance between ploidy and cytoplasmic volume, rather than from haploidy itself. Haploid and double-sized diploid embryos exhibit spindle and contractile ring defects, resulting in chromosome nondisjunction and cytokinesis failure beginning at the second mitosis. Reducing cytoplasmic volume to restore the DNA-to-cytoplasm ratio rescues these abnormalities. Delaying the reduction of the DNA-to-cytoplasm ratio until the late 2-cell stage prevents developmental defects, revealing a critical temporal window for this ratio. Moreover, moderate transcriptional inhibition at this stage in normal diploid embryos induces similar errors. Our findings highlight that in mammalian embryos, where major zygotic genome activation occurs in large cells, balance between ploidy and cytoplasmic volume is necessary to sustain sufficient protein levels for mitosis and development.

## Highlights

• Halved D/C ratio underlies poor blastocyst development in haploid mouse embryos
• Mitotic defects arise after the 2nd cleavage under halved D/C ratio
• Delaying D/C ratio halving until late 2-cell stage alleviates mitotic defects
• Transcriptional insufficiency at ZGA contributes to halved-D/C-related failure

## Graphical abstract

**Figure:**
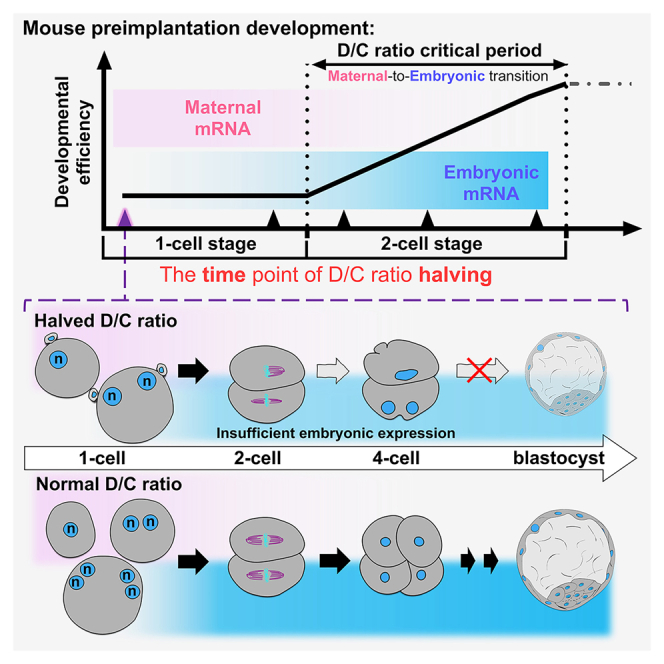

## Introduction

In typical sexual reproduction in animals, embryonic development begins with the fusion of a sperm and an oocyte. Since both gametes complete meiosis before fertilization, the embryo starts with the same ploidy as the parents. However, oocytes can occasionally initiate development without fertilization, either spontaneously or in response to artificial stimulation. These parthenogenetic embryos begin development with half the parental ploidy, though their developmental outcomes vary widely. In Hymenoptera such as bees and ants, fertilized eggs develop into females, while parthenogenetic embryos develop into males, showing that both diploid and haploid embryos can complete development.^1^ In contrast, haploid embryos in vertebrates generally arrest during development, although the timing varies among species. Haploid zebrafish show no major defects before organogenesis compared to diploids,^2^ and haploid *Xenopus laevis*^3^ and *Xenopus tropicalis*^4^ can progress to the neurula and tailbud stages. In mammals, by contrast, haploid embryos exhibit abnormalities at a much earlier stage. Most arrest before reaching the blastocyst stage, suggesting that developmental failure occurs within five to six cell divisions.^5^^,^^6^^,^^7^ Since diploid parthenogenetic embryos achieve preimplantation development comparably to fertilized embryos,^5^ the impaired development of haploid mouse embryos is unlikely to result solely from epigenetic differences between maternal and paternal genomes.^8^ Instead, it may reflect mammal-specific regulatory mechanisms that remain unclear.

In addition to genome ploidy, cell size also influences early embryonic development. Since mammalian embryos gain direct access to maternal resources after implantation, oocytes do not require extensive cytoplasmic nutrient storage, resulting in relatively smaller sizes compared to non-mammalian oocytes,^9^ typically ranging from 60 to 120 μm in diameter.^10^ However, sufficient cytoplasmic volume is still required to support early development. Experimental studies show that artificially reducing mouse oocyte volume significantly impairs post-fertilization developmental potential.^11^^,^^12^ Larger oocytes are often associated with better maturation and higher developmental efficiency *in vitro.*^13^ However, a larger cell volume is not always advantageous. Increased cytoplasmic volume can dilute checkpoint signals at kinetochores, weakening the spindle assembly checkpoint.^12^^,^^14^ It can also hinder proper acentrosomal spindle assembly, increasing the risk of chromosome segregation errors and aneuploidy.^12^ Although this has not yet been confirmed in preimplantation embryos,^15^ early studies in mice showed that artificially enlarging diploid zygotes reduces preimplantation developmental potential.^16^^,^^17^ These findings suggest that early mammalian embryo size is constrained within an optimal range, but the underlying mechanisms remain unclear.

Among cell size-related factors, the DNA-to-cytoplasm (D/C) ratio has drawn particular attention. It remains relatively stable within specific cell types and is closely linked to biosynthetic capacity and transcriptional regulation. A persistently low D/C ratio, as seen in enlarged cells, has been suggested as being associated with cancer and senescence.^18^^,^^19^^,^^20^ In early embryonic blastomeres, unlike somatic cells, cytoplasmic volume does not increase during cleavage divisions, leading to a progressive rise in the D/C ratio.^21^ In fast-developing non-mammalian vertebrates such as *Xenopus* and zebrafish, the D/C ratio contributes to triggering zygotic genome activation (ZGA).^22^^,^^23^^,^^24^^,^^25^ Experimental manipulation, either by increasing DNA or reducing cytoplasm, shows that an elevated D/C ratio accelerates ZGA, whereas a reduced ratio delays it.^26^^,^^27^^,^^28^ These timing changes, however, do not necessarily impair early development. In mice, altering the D/C ratio does not appear to affect the timing of major ZGA.^29^

In this study, we show that the poor developmental success of mouse haploid embryos results primarily from a halved D/C ratio. Stage-specific manipulations revealed a previously unrecognized temporal window during the 2-cell stage, in which a reduced D/C ratio leads to insufficient concentrations of critical proteins expressed during major ZGA. Consequently, mitotic stability is compromised from the 2-cell stage onward, ultimately resulting in preimplantation developmental arrest.

## Results

### Halving the cytoplasmic volume in haploid embryos improves preimplantation development

**Figure 1:**
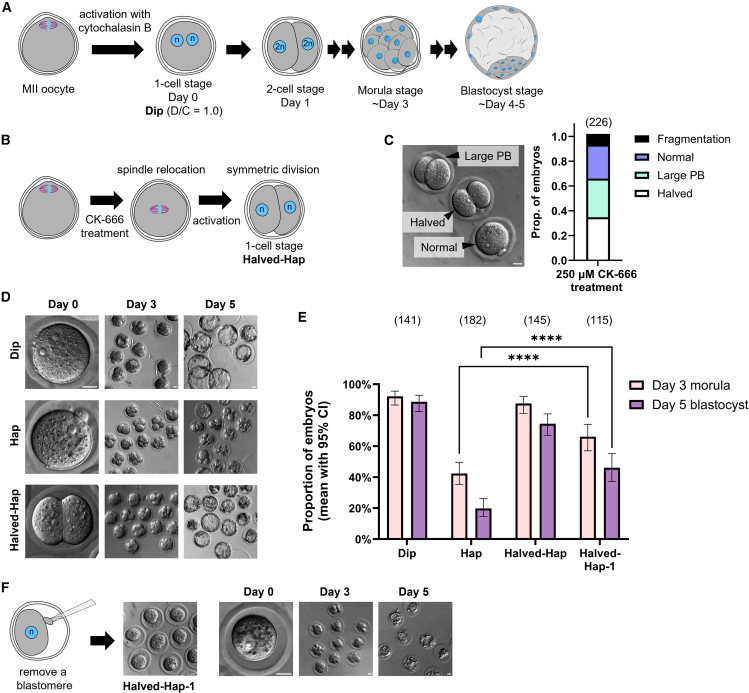
Half-sized haploid embryos exhibit improved preimplantation development compared to normal-sized haploid embryos (A) Schematic of preimplantation development of normal-sized diploid embryo (Dip). (B) Schematic of half-sized haploid embryo (Halved-Hap) production. See also Figure S1C. (C) Bright-field image and bar graph showing proportions of embryos treated with 250 μM CK-666 (mean; five experiments). (D) Bright-field images of Dip, normal-sized haploid (Hap), and Halved-Hap cultured *in vitro*. (E) Proportions of embryos reaching the morula (day 3) and blastocyst (day 5) stages (mean with Wilson/Brown 95% CI; ≥6 experiments). Pairwise comparisons were assessed by two-sided Fisher’s exact tests, p < 0.0001 (∗∗∗∗). (F) Representative images of Halved-Hap-1 production and *in vitro* culture. Scale bars, 20 μm. Sample sizes are shown in parentheses above each bar.

Since activated oocytes and early cleavage-stage blastomeres maintain similar sizes regardless of ploidy, the D/C ratio in haploid embryos is about half that of diploids. Thus, when the D/C ratio of a diploid embryo (Dip) is set to 1.0, that of a haploid embryo (Hap) is 0.5 (Figures 1A, S1A, and S1B). We hypothesized that the reduced D/C ratio, rather than haploidy itself, primarily accounts for the lower developmental potential in Hap. To test this, we established a non-invasive method to induce symmetric division at the end of meiosis II, replacing the normal asymmetric division that extrudes the second polar body, to generate haploid embryos with halved cytoplasmic volume. Treating Metaphase-II (Meta-II) oocytes with CK-666, an Arp2/3 inhibitor, causes spindle relocation from the cortex to the center^30^ (Figures 1B and S1C). Sequential treatment with CK-666 and parthenogenetic activation stimuli yielded nearly symmetric division in ∼30% of activated oocytes, producing half-sized haploid embryos (Halved-Hap; Figure 1C).

Dip, Hap, and Halved-Hap were cultured for 5 days with imaging every 24 h, and the proportions of embryos that developed to the morula (∼72 hpa, day 3) and blastocyst (∼120 hpa, day 5) stages were quantified (Figures 1D and 1E). Notably, despite being haploid, over 70% of Halved-Hap embryos reached the blastocyst stage by day 5, a significantly higher proportion compared to Hap (Figure 1E). They began as two equivalent cells, resulting in twice the cell number of Hap, which may confer a developmental advantage. To rule out this advantage, one blastomere was removed by aspiration to generate embryos hereafter referred to as Halved-Hap-1 (Figure 1F), which still developed more efficiently than Hap (Figure 1E). Furthermore, fusing the two blastomeres of 2-cell stage Hap via electrostimulation produced a single blastomere with a diploid genome but a halved D/C ratio relative to 2-cell stage Dip (Figure S1D). Despite being diploid, these embryos exhibited low developmental efficiency (Figures S1E and S1F). Together, these findings suggest that the D/C ratio, rather than ploidy, is the key determinant of preimplantation development in haploid embryos.

### Haploid embryos tend to exhibit a prolonged 2-cell stage and mitotic abnormalities at the second and third division

**Figure 2:**
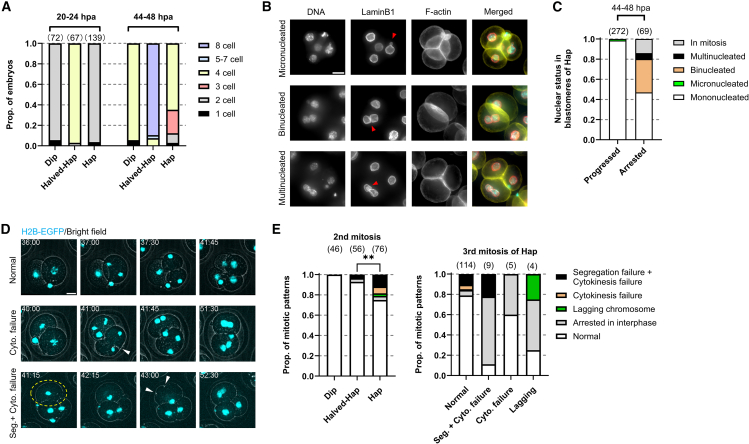
Normal-sized haploid embryos show abnormalities from the second mitosis (A) Proportions of embryo statuses at 20–24 and 44–48 h post-activation (hpa). Statuses are represented by cell number rather than developmental stage (mean; ≥3 experiments). (B) Z-projected immunofluorescence images of Hap at 44–48 hpa. Red arrows indicate nuclei with abnormal morphologies. In merged images, DNA, Lamin B1, and F-actin are shown in cyan, red, and yellow, respectively. (C) Proportion of nuclear status of Hap blastomeres that completed cytokinesis (progressed) or remained 2-cell-sized (arrested) at 44–48 hpa (mean; ≥3 experiments). (D) Still images from time-lapse imaging of the second mitosis in Hap expressing Histone H2B-EGFP. Timestamps indicate hpa. White arrows, incomplete cleavage furrows; yellow dashed lines, blastomeres with segregation and cytokinesis failure; red arrows, micronuclei. See also Video S1. (E) Proportion of mitotic patterns obtained by time-lapse imaging (mean; ≥3 experiments). Pairwise comparisons of proportion of normal blastomeres used two-sided Fisher’s exact tests, p < 0.01 (∗∗). Scale bars, 20 μm. Sample sizes in parentheses above each bar.

Since Hap exhibited significantly lower developmental efficiency before the morula stage compared to Dip, Halved-Hap, and Halved-Hap-1 (Figure 1D), we next examined abnormalities in Hap that might contribute to developmental arrest. Bright-field microscopy revealed that over 95% of Dip, Hap, and Halved-Hap completed the first cleavage and reached the 2-cell stage by 24 hpa. However, by 48 hpa, more than 30% of Hap remained at the 2- or 3-cell stage, whereas over 90% of Dip and Halved-Hap reached the 4-cell stage (Figure 2A). Immunofluorescence at 44–48 hpa showed that nearly 40% of the arrested Hap blastomeres contained two or more nuclei (Figures 2B and 2C), suggesting increased susceptibility to errors during the second mitosis.

To further examine cell cycle and mitotic progression, Dip, Hap, and Halved-Hap embryos expressing H2B-EGFP from Rosa26 locus were subjected to time-lapse imaging. Consistent with immunofluorescence imaging, over 95% completed the first mitosis (Figure S2A). Most Hap entered the second mitosis (Figure 2E) but with several hours of delay compared to Dip and Halved-Hap (Figure S2B). Hap also exhibited prolonged mitosis (Figure S2C), and over 20% of blastomeres showed defects such as severe chromosome segregation errors, including nondisjunction, as well as lagging chromosomes and cytokinesis failure. These abnormalities resulted in either polyploid mononucleated cells or cells containing two or more nuclei (Figures 2D and 2E; Video S1). Notably, when cytokinesis fails during the second mitosis, the cell number does not increase, producing diploid blastomeres that remain 2-cell sized but developmentally correspond to the 4-cell stage, thereby retaining a halved D/C ratio. Cytokinesis failure was often characterized by a cleavage furrow that formed but regressed without completing division (Figure 2D, white arrows). In contrast, Dip and Halved-Hap showed significantly fewer abnormalities (Figure 2E, left). Among Hap blastomeres with second mitosis defects, the majority either arrested or exhibited additional abnormalities, whereas only a minority progressed normally. (Figure 2E, right). Even among those cells completing the second mitosis, 21.1% showed defects in the third (Figure 2E, right). These results indicate that reduced D/C ratio compromises mitotic stability from the second mitosis onward, leading to cumulative defects and developmental arrest in Hap.

Video S1. Haploid embryos exhibit mitotic abnormalities from the second mitosis onward, related to Figure 2

### Haploid embryos with cytokinesis failure exhibit abnormal spindle and contractile ring formation

To investigate the cause of cell cycle delay and mitotic abnormalities in Hap, we first examined whether DNA damage was involved. However, γH2AX foci were rarely detected in Hap nuclei during G2 of the 2-cell stage (Figures S3A and S3B).

**Figure 3:**
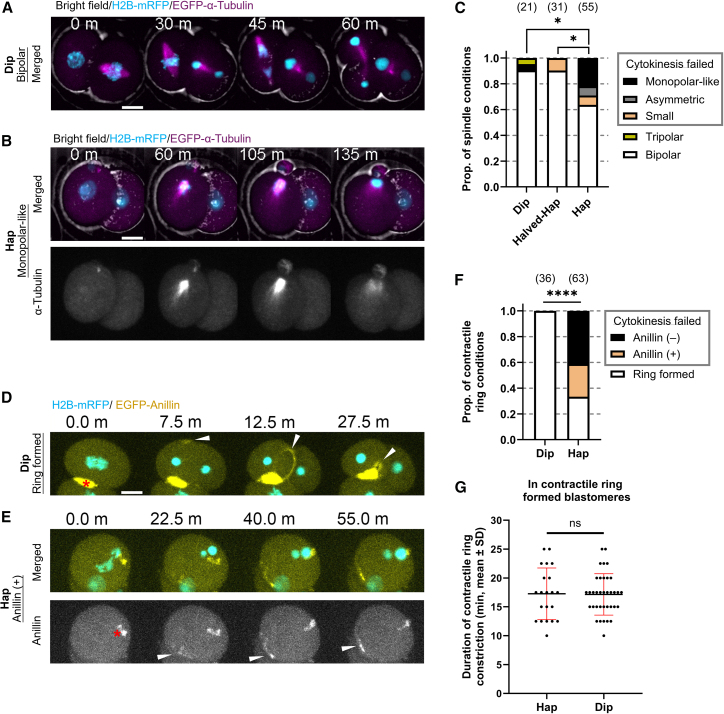
Normal-sized haploid embryos show defects of spindle and contractile ring formation (A and B) Still images from time-lapse imaging of the second mitosis in microinjected embryos expressing histone H2B-mRFP1 and EGFP-α-tubulin. Timestamps indicate time (minutes) since NEBD. See also Video S2. (C) Proportion of spindle conditions during second mitosis in microinjected embryos (mean; ≥3 experiments). Proportion of bipolar spindles was assessed using Fisher’s exact test. (D and E) Still images from time-lapse imaging of the second mitosis in microinjected embryos expressing histone H2B-mRFP1 and EGFP-Anillin. White arrows, accumulation of EGFP-Anillin signals appearing after anaphase; red asterisks, aggregates of EGFP-Anillin signals present on the cell membrane from interphase. Timestamps indicate time (minutes) since anaphase. See also Video S3. (F) Proportion of contractile ring conditions during second mitosis in microinjected embryos (mean; ≥4 experiments). Proportion of ring formed blastomeres was assessed using Fisher’s exact test. (G) Scatterplots showing the time of contractile ring constriction in blastomeres with completed cytokinesis (mean ± SD; *n* ≥ 21; ≥4 experiments). Statistical analysis was performed using unpaired two-tailed *t* test. Scale bars, 20 μm. Sample sizes in parentheses above each bar. Statistical significance is denoted as follows: p < 0.05 (∗), p < 0.0001 (∗∗∗∗); n.s., not significant.

We then assessed spindle formation in the second mitosis by visualizing microtubules through mRNA injection. In the vast majority of Dip and Halved-Hap that entered mitosis, bipolar spindles were formed, and chromosome alignment, segregation, and cytokinesis proceeded normally (Figures 3A and S3C). In contrast, Hap exhibited a higher frequency of mitotic abnormalities, including three major types of spindle abnormalities associated with impaired chromosome dynamics. The most common defect was the formation of monopolar-like spindles, where chromosomes eventually moved toward a single pole and formed one nucleus (Figures 3B and 3C; Video S2). Asymmetric bipolar spindles were also observed, resulting in lagging chromosomes and multinucleated cells (Figure S3D). In the remaining cases, bipolar spindles formed, and segregation appeared normal, but cytokinesis still failed; these spindles tended to be small and occasionally bent (Figure S3E).

Video S2. Spindle structures within defective blastomeres were also aberrant during the second mitosis, related to Figure 3

We further evaluated contractile ring formation by visualizing anillin. Consistently, Hap exhibited a significantly higher frequency of contractile ring abnormalities than Dip (Figures 3D–3F). Among Hap blastomeres that failed cytokinesis, 61.9% exhibited membrane deformation at one or two sites after anaphase, accompanied by anillin accumulation (Anillin +; Figure 3E; Video S3). In the remaining cases, no obvious membrane deformation or anillin accumulation was observed (Anillin −; Figure S3F; Video S3). Nevertheless, in blastomeres that successfully completed cytokinesis, the duration of contractile ring constriction did not differ significantly between Hap and Dip (Figure 3G).

Video S3. Contractile ring formation within defective blastomeres were also aberrant during the second mitosis, related to Figure 3

These findings imply that defective spindle assembly and impaired contractile ring formation contribute to abnormal chromosome dynamics and cytokinesis failure, which may in turn underlie the developmental arrest observed in Hap.

Additionally, under comparable live-cell imaging conditions, exogenous expression of EGFP-tubulin or EGFP-anillin in Hap embryos led to a markedly higher frequency of mitotic abnormalities during the second mitosis, whereas Dip and Halved-Hap embryos remained largely unaffected (Figures 2E, 3C, and 3F), These observations suggest that Hap exhibited lower tolerance to exogenous gene expression and increased susceptibility to mitotic errors.

### Halving the D/C ratio of diploid embryos by doubling the cytoplasmic volume induces developmental defects similar to haploid embryos

**Figure 4:**
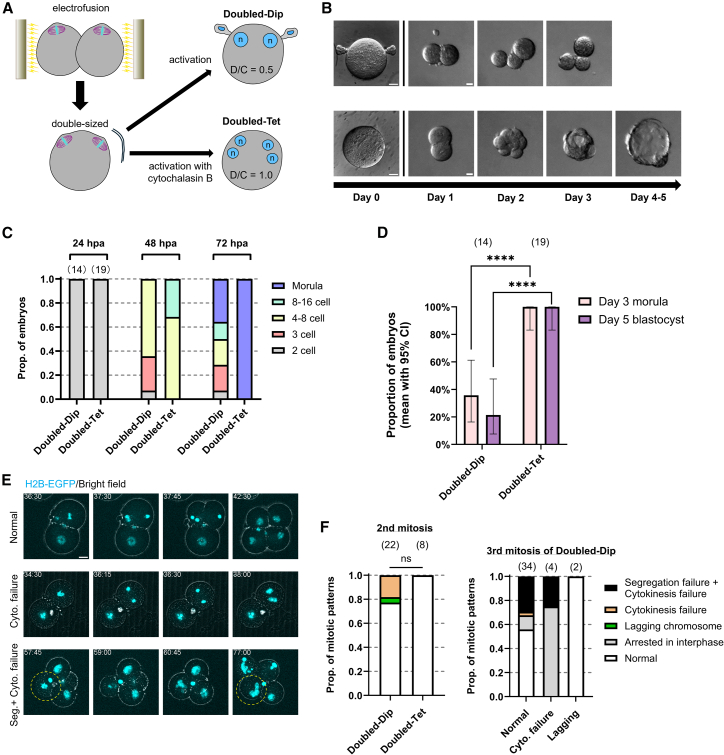
Doubled-sized diploid embryos show developmental defects similar to normal-sized haploid (A) Schematic of double-sized diploid (Doubled-Dip) and tetraploid (Doubled-Tet) embryos production. (B) Bright-field images of Doubled-Dip (top) and Doubled-Tet (bottom) cultured *in vitro*. (C) Proportions of embryo statuses at 24, 48, and 72 hpa (mean; ≥3 experiments). (D) Proportions of embryos reaching morula (day 3) and blastocyst (day 5) stages (mean with Wilson/Brown 95% CI; ≥4 experiments). Pairwise comparisons were assessed by two-sided Fisher’s exact tests. (E) Still images from time-lapse imaging of the second mitosis in Doubled-Dip expressing histone H2B-EGFP. Timestamps indicate hpa. Yellow dashed lines, Segregation failure + Cytokinesis failure blastomeres. See also Video S4. (F) Proportion of mitotic patterns obtained by time-lapse imaging (mean; Doubled-Dip from 5 experiments, Doubled-Tet from 2 experiments). Proportion of normal blastomeres was assessed using Fisher’s exact test. Scale bars, 20 μm. Sample sizes in parentheses above each bar. Statistical significance is denoted as follows: p < 0.0001 (∗∗∗∗); n.s., not significant.

To further investigate the importance of the D/C ratio, we generated diploid and tetraploid embryos with doubled cytoplasmic volume by electrofusing two Meta-II oocytes (Figures 4A and S4A). Electrostimulation induced simultaneous fusion and activation, resulting in diploid embryos with 2 s polar bodies and a relative D/C ratio of 0.5 (Doubled-Dip). When cytochalasin B was added to inhibit polar body extrusion, tetraploid embryos were generated with the same cytoplasmic volume and a relative D/C ratio of 1.0 (Doubled-Tet) (Figures 4A, S1A, and S1B).

As expected, about 40% of Doubled-Dip, despite completing the first division, arrested at the 2- or 3-cell stage, and only 35.7% and 21.4% reached the morula and blastocyst stages, respectively (Figures 4B–4D). Immunofluorescence revealed binucleated blastomeres in embryos arrested before the 8-cell stage, similar to Hap (Figure S4B). In contrast, all 19 Doubled-Tet reached the blastocyst stage (Figure 4D).

Live-cell imaging revealed that Doubled-Dip exhibited cell cycle and mitotic abnormalities similar to those in Hap. They entered the second mitosis after several hours of delay and showed prolonged mitosis compared to Doubled-Tet (Figures S4C and S4D). Moreover, although the small sample size precluded statistical significance, Doubled-Dip showed a higher incidence of abnormalities from the second mitosis (Figures 4E and 4F; Video S4), mainly cytokinesis failure leading to binucleated blastomeres. As in Hap, blastomeres of Doubled-Dip that doubled their ploidy due to cytokinesis failure during the second mitosis did not improve stability in the third mitosis. Instead, they exhibited chromosome segregation errors or underwent developmental arrest. Even among blastomeres that completed the second mitosis normally, more than 40% still experienced abnormalities in the third (Figure 4F). Together, these findings indicate that halving the D/C ratio significantly impairs preimplantation development, even in diploid embryos. Embryos with a relative D/C ratio of 0.5 consistently showed a prolonged 2-cell stage and frequent mitotic defects, primarily cytokinesis failure starting at the second cleavage.

Video S4. Diploid embryos with doubled cytoplasmic volume exhibit mitotic abnormalities from the second mitosis onward, related to Figure 4

### Developmental defects also occur when the D/C ratio is halved at the late G2 phase of the 1-cell stage

**Figure 5:**
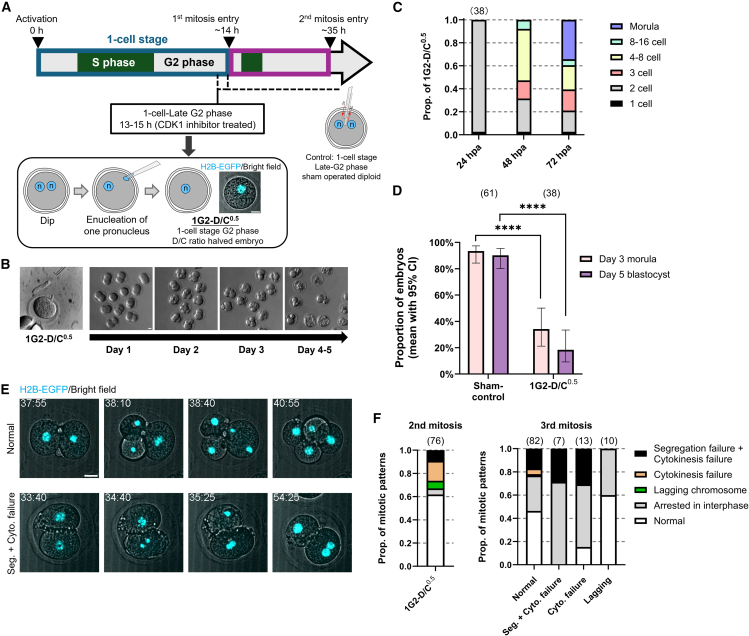
Postponing D/C ratio halving from the second meiosis to late G2 phase in 1-cell stage fails to rescue development (A) Schematic of experimental design. Bottom right: Representative still image from time-lapse imaging of 1-cell stage G2 phase D/C ratio halved embryo (1G2-D/C^0.5^). (B) Bright-field images of 1G2-D/C^0.5^ after enucleation (left) and *in vitro* culture. (C) Embryo statuses at 24, 48, and 72 hpa (mean; ≥4 experiments). (D) Proportions of embryos reaching the morula (day 3) and blastocyst (day 5) stages (mean with Wilson/Brown 95% CI; ≥4 experiments). Pairwise comparisons were assessed by two-sided Fisher’s exact tests, p < 0.0001 (∗∗∗∗). (E) Still images from time-lapse imaging of the second mitosis in 1G2-D/C^0.5^ expressing histone H2B-EGFP. Timestamps indicate hpa. See also Video S6. (F) Proportion of mitotic patterns of 1G2-D/C^0.5^ obtained by time-lapse imaging (mean; ≥3 experiments). Scale bars, 20 μm. Sample sizes are shown in parentheses above each bar.

In previous experiments, we manipulated the D/C ratio at the onset of development. To determine whether a proper D/C ratio is required throughout preimplantation development or only during specific stages, we next reduced the D/C ratio at later time points. During cleavage, embryonic cells divide without growing, so the D/C ratio is lowest at the 1-cell stage and doubles with each division. A relative D/C ratio of 0.5 at the 1-cell stage is thus not normally encountered and may impair progression. To examine whether the unusually low D/C ratio at the 1-cell stage impairs development, Dip was cultured to the late G2 phase of the first cell cycle, and one of the two pronuclei was removed, generating haploid embryos (hereafter 1G2-D/C^0.5^; Figure 5A). To ensure enucleation just before the first cleavage, which begins around 14 hpa, mitotic entry was blocked with a CDK1 inhibitor, and enucleation was performed between 13 and 15 hpa. Upon inhibitor release, over 90% of the embryos entered mitosis and reached the 2-cell stage without detectable mitotic defects (Figures 5B, 5C, and S5A; Video S5). Sham control embryos, subjected to identical treatment and microneedle insertion but without enucleation, developed efficiently to the blastocyst stage. In contrast, 1G2-D/C^0.5^ exhibited a trajectory resembling that of Hap. By day 2, approximately 45% arrested at the 2–3-cell stage, and only 18.4% formed blastocysts (Figures 5C and 5D). Live-cell imaging further revealed delayed entry into the second mitosis, prolonged mitotic progression (Figures S5B and S5C), and mitotic abnormalities similar to those seen in Hap and Doubled-Dip (Figures 5E and 5F; Video S6).

Video S5. The first mitosis of 1G2-D/C^0.5^ proceeds without abnormalities, related to Figure S5

Video S6. The second mitosis of 1G2-D/C^0.5^ exhibits mitotic abnormalities, related to Figure 5

Notably, nuclei in 2-cell-stage blastomeres of 1G2-D/C^0.5^ were smaller than those in Dip or Hap embryos (Figures S5D and S5E). Although nuclear size did not differ significantly between blastomeres with or without mitotic abnormalities in the subsequent division (Figure S5F), reduced nuclear size may still have contributed to impaired mitosis and lower developmental potential.

### Postponing the reduction of the D/C ratio to the late 2-cell stage circumvents developmental defects

**Figure 6:**
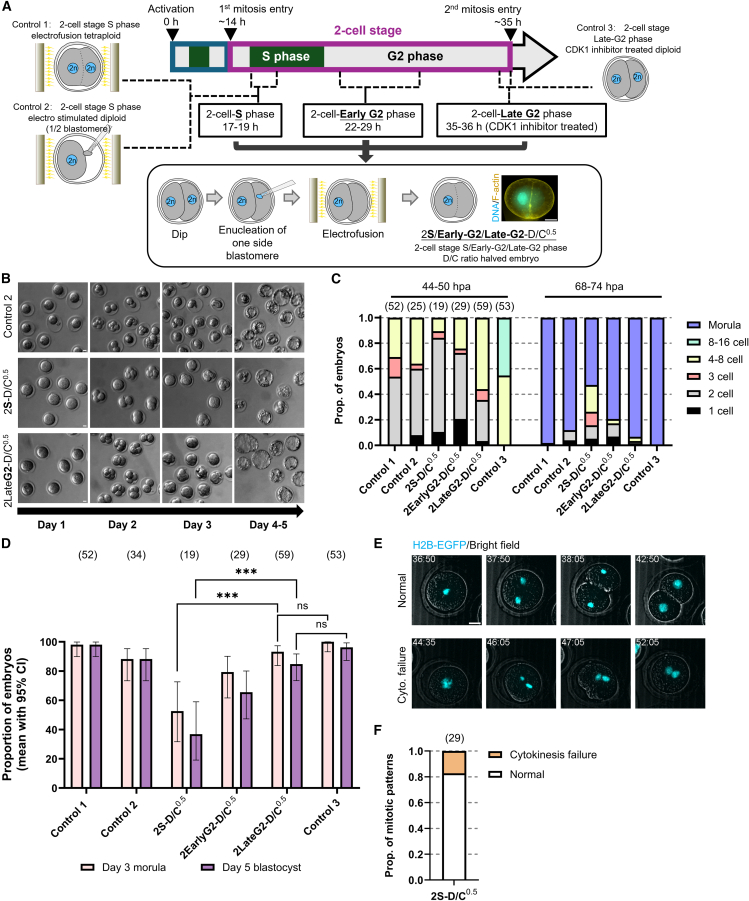
Postponing D/C ratio halving from the 1-cell stage to the 2-cell stage alleviates developmental defects (A) Schematic of experimental design. Bottom right: representative immunofluorescence image of a 2-cell stage S phase D/C ratio halved embryo (2S-D/C^0.5^). (B) Bright-field images of control 2 embryos (as described in A, top), 2S-D/C^0.5^ and 2-cell stage late G2 phase D/C ratio halved embryo (2LateG2-D/C^0.5^) cultured *in vitro*. (C) Embryo statuses at 24, 48, and 72 hpa (mean; ≥3 experiments). (D) Proportions of embryos reaching morula (day 3) and blastocyst (day 5) stages (mean with Wilson/Brown 95% CI; ≥3 experiments). Pairwise comparisons were assessed by two-sided Fisher’s exact tests, p < 0.001 (∗∗∗); n.s., not significant. (E) Still images from time-lapse imaging of the second mitosis in 2S-D/C^0.5^ expressing histone H2B-EGFP. Timestamps indicate hpa. See also Video S7. (F) Proportion of second mitosis patterns in 2S-D/C^0.5^ obtained by time-lapse imaging (mean; 3 experiments). Scale bars, 20 μm. Sample sizes are shown in parentheses above each bar.

To evaluate the effects of reducing the D/C ratio at later developmental stages without altering nuclear size, we halved the D/C ratio at several time points during the 2-cell stage. This was achieved by enucleating one blastomere and fusing it with the other to form a single cell (Figure 6A). Cell cycle progression in 2-cell-stage Dip embryos was determined by 5-bromo-2′-deoxyuridine (BrdU) incorporation (Figures S5G–S5H). Based on this, the D/C ratio was reduced during early S phase (17–19 hpa), early G2 phase (22–29 hpa), and late G2 phase (35–36 hpa, following CDK1 inhibition), generating 2S-D/C^0.5^, 2EarlyG2-D/C^0.5^, and 2LateG2-D/C^0.5^, respectively (Figure 6A). To account for possible procedural effects, three control groups with a relative D/C ratio of 1.0 were designed and established. These included: (control 1) electrofusion of two blastomeres to generate tetraploid embryos at the early S phase; (control 2) removal of one blastomere followed by electrical stimulation of the other; and (control 3) 2-cell stage Dip treated with CDK1 inhibitor in the late G2 phase (Figure 6A).

All control embryos efficiently developed to the blastocyst stage (Figures 6B–6D), indicating that the procedures themselves did not significantly affect development. In contrast, 2S-D/C^0.5^ frequently arrested before the morula stage, with 52.6% reaching the morula and 36.8% the blastocyst stage. These rates were modestly higher than those of Hap and Doubled-Dip, but still significantly lower than embryos with a relative D/C ratio of 1.0 (Figure 6D). Moreover, live-cell imaging revealed that approximately 20% of 2S-D/C^0.5^ failed to complete cytokinesis during the second mitosis (Figures 6E and 6F; Video S7), resembling defects observed in other halved D/C ratio embryos such as Hap and Doubled-Dip. These findings indicate that even when the D/C ratio is normal during the 1-cell stage, its reduction from the early 2-cell stage can induce defects starting at the second mitosis, ultimately leading to developmental arrest. Importantly, developmental efficiency improved as the timing of D/C ratio halving was delayed within the 2-cell stage, reaching control levels in 2LateG2-D/C^0.5^ (Figure 6D). This suggests that maintaining a normal D/C ratio until the end of the 2-cell stage is critical, whereas reductions at later stages do not appear to impair preimplantation development.

Video S7. The second mitosis of 2S-D/C^0.5^ exhibits mitotic abnormalities, related to Figure 6

### Reduced transcript levels at the 2-cell stage may be one of the causes of the D/C ratio-related abnormalities

Considering that the 2-cell stage is a critical period for the D/C ratio and coincides with major ZGA in mouse embryos, we hypothesized that the developmental defects observed in embryos with a halved D/C ratio might result from insufficient concentrations of transcriptional products during this stage. To address this, we first employed the 5-ethynyl uridine (EU)-Click assay to assess nascent RNA in Hap and Dip embryos at the 2-cell stage. 1-h incorporation assays at multiple time points revealed that Hap had a shorter transcriptional peak duration and greater inter-embryo variability compared to Dip (Figures S6A–S6C). Furthermore, a 20–30 hpa EU incorporation experiment indicated that overall transcriptional output in Hap was lower than in Dip (Figures S6A and S6D).

**Figure 7:**
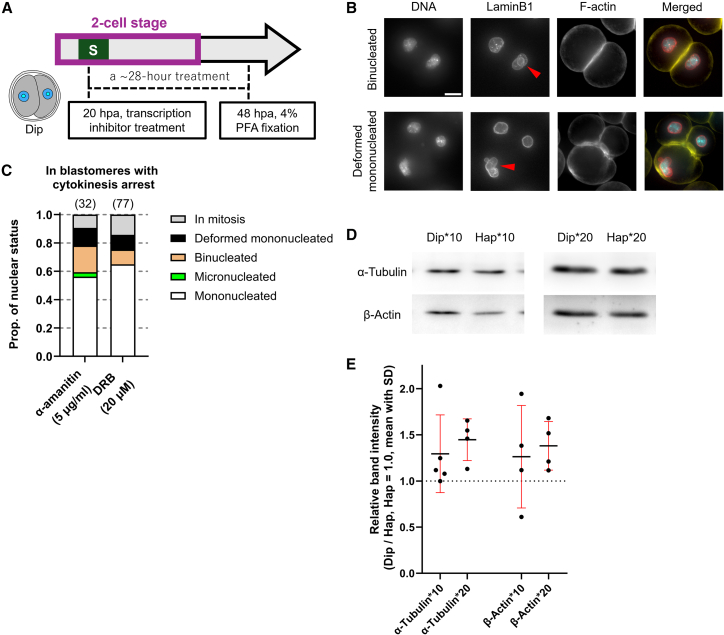
Mild transcriptional inhibition at the 2-cell stage induces mitotic instability in diploid embryos (A) Schematic of experimental design. (B) Z-projected immunofluorescence images of transcriptional inhibited Dip at 44–48 hpa. Red arrows indicate nuclei with abnormal morphologies. In merged images, DNA, Lamin B1, and F-actin are shown in cyan, red, and yellow, respectively. Scale bars, 20 μm. (C) Proportion of nuclear status of blastomeres that remained 2-cell-sized (arrested) after transcriptional inhibition (mean; 3 experiments). Sample sizes are shown in parentheses above each bar. (D) Representative immunoblots of α-tubulin and β-actin. Lanes were loaded with lysates from 10 or 20 2-cell stage embryos. (E) Quantification of immunoblot signals. Each dot represents the intensity ratio of a paired Dip and Hap band (mean ± SD). For experiments using lysates from 10 embryos, *n* ≥ 4 (4 experiments); for experiments using lysates from 20 embryos, *n* = 4 (2 experiments).

We next tested whether low-dose transcriptional inhibition in Dip embryos could induce Hap-like abnormalities. To mimic the timing of D/C ratio reduction in 2S-D/C0.5 (Figure 6A), Dip were treated with transcriptional inhibitors from 20 hpa through the second mitosis (Figure 7A). Treatment with low to moderate concentrations of α-amanitin or 5,6-dichloro-1-β-D-ribofuranosylbenzimidazole (DRB) did not completely block development (Figures S6E and S6F). Although most blastomeres advanced to the 4-cell stage, a subset remained arrested with a 2-cell-like morphology. Immunofluorescence staining revealed that these arrested blastomeres were either binucleated or contained deformed mononuclei (Figures 7B and 7C), partially recapitulating the phenotypes observed in Hap (Figures 2B and 2C). These findings raise the possibility that the observed abnormalities were likewise caused by defective chromosome segregation and cytokinesis. We further examined the levels of two key proteins involved in spindle and contractile ring formation. Western blot analysis at 30 hpa revealed that α-tubulin and β-actin levels were lower in Hap embryos than in Dip embryos at the late 2-cell stage (Figures 7D–7E).

Taken together, our results suggest that the essential role of the D/C ratio is to maintain a balance between the genome’s transcriptional capacity and cytoplasmic volume during the major ZGA at 2-cell stage. When cell volume is disproportionately large relative to the available transcriptional templates, the concentrations of certain embryonic factors decline, leading to mitotic abnormalities and increased risk of developmental arrest.

## Discussion

In placental mammals, embryos with a diploid genome of uniparental origin, or a biparental genome in a non-diploid state, fail to support post-implantation development.^31^^,^^32^^,^^33^^,^^34^^,^^35^ In contrast, these constraints do not apply during preimplantation development. Diploid embryos with only maternal or paternal genomes, as well as polyploid embryos, can develop efficiently to the blastocyst stage.^5^^,^^36^ However, haploid embryos, whether of maternal or paternal origin, exhibit markedly low developmental efficiency, with most arresting before the blastocyst stage for reasons that remain unclear. In this study, by generating embryos with varying cell sizes and genome ploidies, we show that while haploidy may contribute to developmental failure, the primary cause of arrest is an imbalance between genomic DNA content and cytoplasmic volume.

Cytoplasmic aspiration with a glass capillary is a standard method to reduce the size of mouse oocytes and early embryos.^15^^,^^29^^,^^37^^,^^38^ Here, we instead used a non-invasive approach that alters spindle positioning to convert the second meiotic division from asymmetric to symmetric, thereby reducing cytoplasmic volume in one-cell embryos. We analyzed embryos that divided nearly equally, yielding cells with about half the original volume (Figure S1A). As this method also produces embryos with varying asymmetry, it can generate a range of D/C ratios.

The higher developmental efficiency of Halved-Hap and Halved-Hap-1 compared to Hap suggests that although diploidy and oocyte size are considered important for preimplantation development, maintaining the balance between genomic content and cytoplasmic volume can alleviate developmental impairment (Figure 1E). Nevertheless, their developmental efficiencies remain lower than that of Dip, indicating that haploidy may impose additional limitations even when the D/C ratio is normalized. The higher blastocyst formation efficiency in Halved-Hap compared to Halved-Hap-1 implies that starting development with a greater number of blastomeres may help offset reduced proliferation associated with haploidy. Moreover, despite diploidy, Doubled-Dip exhibited low blastocyst formation efficiency. These efficiencies were restored by inducing tetraploidy, indicating that simply increasing cytoplasmic volume does not improve preimplantation development (Figure 4D). Rather, this highlights the importance of maintaining a proper balance between genome content and cytoplasmic volume. Similar findings have been reported in studies using embryos produced by fusing multiple oocytes, where increased cytoplasmic volume was associated with reduced developmental success.^16^^,^^39^

The primary developmental barrier in Hap arises before the morula stage, beginning at the 2-cell stage. Although over 30% of Hap appear morphologically arrested at the 2-cell stage (Figure 2A), live imaging showed that nearly all blastomeres had entered the second mitosis, despite a delayed interphase (Figure 2F). However, many failed to complete cytokinesis, with or without preceding chromosome segregation failure, and thus retained a 2-cell-like appearance. Cytokinesis failure has also been reported in haploid somatic cells, often resulting in diploidization. Notably, diploidized somatic cells exhibit greater stability and proliferative capacity than haploid cells.^40^^,^^41^ In contrast, most blastomeres in Hap embryos undergoing cytokinesis failure either arrested in the next cell cycle or accumulated further mitotic errors (Figure 2E). These differences suggest that the mechanisms of cytokinesis failure differ between Hap blastomeres and haploid somatic cells, which appear to maintain a D/C ratio similar to diploid cells.^42^ In Hap blastomeres, the defects are more likely caused by a reduced D/C ratio, as similar abnormalities were observed in Doubled-Dip.

Knoblochova et al. reported that shortening of the 2-cell stage in mouse embryos compromises genome integrity and leads to genome fragmentation during subsequent divisions,^43^ resembling the multinucleation we observed from the asymmetric spindle (Figure S3D). This initially led us to suspect that reducing the D/C ratio might cause similar DNA damage. In contrast, our γH2A.X staining did not detect such effects (Figure S3B), and embryos with a halved D/C ratio in fact displayed a prolonged 2-cell stage (Figures S2B, S4C, and S5C).^43^ Instead, the reduced D/C ratio compromises mitotic stability by causing abnormalities in spindle and contractile ring formation.

The first mitosis, when the cell is at its largest, did not show an increased frequency of abnormalities upon reduction of the D/C ratio (Figures S2A and S5A). This suggests that mitotic errors are not simply caused by an imbalance between DNA content and cell size. A recent study in *Caenorhabditis elegans* demonstrated that although transcription is partially upregulated under low D/C conditions, intracellular mRNA concentrations remain diluted, with highly expressed transcripts being particularly susceptible to this effect.^44^ This is consistent with our result that the level of nascent RNA in Hap at 20–30 hpa was lower than in Dip, though not reduced to half (Figure S6B). Furthermore, previous studies in budding yeast and human cell lines have demonstrated that an abnormally low D/C ratio causes genome dilution, which, in severe cases, leads to a collapse of biosynthetic activity.^18^^,^^19^ Building on this evidence, and together with the results from transcriptional inhibition (Figures 7A–7C), we speculate that in 2-cell stage mouse embryos, certain proteins, such as those involved in spindle and contractile ring assembly, may become limiting when the D/C ratio is halved, due to a reduced number of transcriptional templates relative to cell volume. Indeed, Hap at the late 2-cell stage displayed lower levels of α-tubulin and β-actin compared with Dip (Figure 7E). This hypothesis explains why the first mitosis proceeds normally, when maternal factors remain abundant, and why reducing the D/C ratio later in the 2-cell stage does not impair development, as key embryonic factors have already been produced.

Compared to other vertebrates, mammalian oocytes have a relatively small volume.^9^ However, because ZGA occurs after fewer cell divisions, blastomeres remain considerably large at the time of ZGA.^22^ In addition, ZGA coincides with the degradation of maternal mRNAs.^45^ As a result, a reduced D/C ratio at this stage is expected to cause more severe abnormalities than at other developmental stages or in other vertebrates, as transcriptional output cannot compensate for the large cytoplasmic volume. Binucleated blastomeres with equally sized nuclei are relatively common in human embryos generated by *in vitro* fertilization.^46^^,^^47^^,^^48^^,^^49^^,^^50^ A previous study reported that, in humans, where ZGA occurs at the 8-cell stage, approximately 65% of embryos at the 9- to 16-cell stage contain one to six binucleated blastomeres. A subset of these blastomeres subsequently arrests during later cleavage divisions.^51^ These post-ZGA binucleations may involve mechanisms similar to those underlying the mitotic abnormalities observed in our study, although our use of parthenogenetic embryos means that further validation in fertilized embryos is warranted.

Together, our findings demonstrate that a balanced genome-to-cytoplasm ratio is essential for faithful cell division and developmental progression during the transition from maternal to zygotic control in mammalian embryos. This highlights a critical interplay between cell size and transcriptional capacity and offers new insights into why mammalian embryos are particularly vulnerable to genome imbalance. Further studies will advance our understanding of the mechanisms underlying early embryonic development unique to mammals.

### Limitations of the study

1. All embryonic models used in this study were derived from parthenogenesis and therefore lacked a paternal genome. Further investigation using fertilized zygotes will be important to determine whether our findings are broadly applicable.
2. The resolution of the live-cell imaging system used in this study was limited, which may have affected the accuracy of mitotic abnormality analysis. Future studies employing imaging systems with higher temporal and spatial resolution will likely enable more precise characterization of these abnormalities.

## Resource availability

### Lead contact

Information and requests for resources and reagents should be directed to and will be fulfilled by the lead contact, Miho Ohsugi (mihoohsugi@g.ecc.u-tokyo.ac.jp).

### Materials availability

This study did not generate new unique reagents.

### Data and code availability

• All data reported in this paper is available from the lead contact upon request.
• This paper does not report original code.
• Any additional information required to reanalyze the data reported in this paper is available from the lead contact upon request.

## Acknowledgments

We thank Tomo Kondo and Ryohei Nakamura for valuable discussions and insightful comments on the manuscript. We are grateful to Hiroshi Kimura for providing the plasmid encoding mRFP1-tagged histone H2B, to Kazuo Yamagata for the pcDNA3.1-pA vector and the plasmid encoding EGFP-tagged α-tubulin, and to Ryota Uehara for the plasmid encoding EGFP-Anillin-Cterminal. We also thank Hirohisa Kyogoku and Hiroyuki Imai for their technical advice on enucleation and electrofusion of mouse embryos. This work was supported by the 10.13039/501100001691Japan Society for the Promotion of Science (JSPS) KAKENHI (grant nos. 18K19325, 20H05356, 22H04664, and 23H02485). The first author, T.P., was supported in 2024 by JST SPRING (grant no. JPMJSP2108).

## Author contributions

T.P. and M.O. conceived and designed the study; N.T. and T.P. developed the method to generate half-sized embryos, performed immunofluorescence and live imaging of haploid embryos, and analyzed the corresponding data; T.P. conducted all other experiments and performed the remaining data analyses; T.P. drafted the manuscript; M.O. supervised the project, reviewed and revised the manuscript, and secured funding.

## Declaration of interests

The authors declare no competing interests.

## Declaration of generative AI and AI-assisted technologies in the writing process

The author used ChatGPT (OpenAI, GPT-4, 5) to assist with language refinement, including improvements to clarity, grammar, and readability. All content was critically reviewed and approved by the author, who assumes full responsibility for the final manuscript.

## STAR★Methods

### Key resources table

**Table:** 

REAGENT or RESOURCE	SOURCE	IDENTIFIER
**Antibodies**		

Alpha Tubulin (YOL1/34), Rat mAb	Santa Cruz Biotechnology	sc-53030; RRID: AB_2272440
Anti-TUBA4A (TUBA1), Mouse mAb	Sigma-Aldrich	T9026; RRID:AB_477593
Lamin B1 (B-10) Alexa Fluor 488, Mouse mAb	Santa Cruz Biotechnology	sc-374015; RRID: AB_10947408
phospho-Histone H2A.X (Ser139), Mouse mAb	Merck Millipore	JBW301; RRID: AB_309864
Anti-β-Actin, mAb	MBL	M177-3; RRID:AB_10697039
Anti-BrdU, Rat mAb	BioRad	OBT0030; RRID: AB_2314029
Goat anti-Mouse IgG (H+L) Highly Cross-Adsorbed Secondary Antibody, Alexa Fluor™ 488	Invitrogen	A-11029; RRID: AB_2534088
Goat anti-Mouse IgG (H+L) Highly Cross-Adsorbed Secondary Antibody, Alexa Fluor™ 555	Invitrogen	A-21424; RRID: AB_141780
Goat anti-Mouse IgG (H+L) Highly Cross-Adsorbed Secondary Antibody, Alexa Fluor™ 647	Invitrogen	A-21236; RRID: AB_2535805
Donkey anti-Rat IgG (H+L) Highly Cross-Adsorbed Secondary Antibody, Alexa Fluor™ 488	Invitrogen	A-21208; RRID: AB_141709
Donkey anti-Rat IgG (H+L) Highly Cross-Adsorbed Secondary Antibody, Alexa Fluor™ 568	Invitrogen	A78946; RRID: AB_2910653
Donkey anti-Rat IgG (H+L) Highly Cross-Adsorbed Secondary Antibody, Alexa Fluor™ 647	Invitrogen	A78947; RRID: AB_2910635
Sheep Anti-Mouse IgG - Horseradish Peroxidase	Cytiva	NA931; RRID: AB_772210

**Chemicals, peptides, and recombinant proteins**		

Rhodamine phalloidin	Invitrogen	R415;
Hoechst 33342 dye	Thermo Fisher Scientific	H-1399
Pregnant mare serum gonadotropin (PMSG)	ZENOAQ	N/A
Human chorionic gonadotropin (hCG)	Kyoritsu Seiyaku	N/A
Hyaluronidase from bovine testes	Sigma-Aldrich	H4272
M16 medium	Sigma-Aldrich	M7292
M2 medium	Sigma-Aldrich	M7167
Tyrode’s solution	Sigma-Aldrich	T1788
Cytochalasin B	Sigma-Aldrich	C6762
Ribo m^7^G Cap Analog	Promega	P1711
Paraffin oil	Nacalai Tesque	26137-85
Ro-3306	COSMO BIO	HY-12529
CK-666	Selleck	S7690
Etoposide	WAKO	055-08431
Phytohemagglutinin-P	WAKO	161-15251
BrdU (5-Bromo-2'-deoxyuridine)	Nacalai Tesque	05650-24
α-Amanitin	WAKO	010-22961
DRB (5,6-Dichlorobenzimidazole 1-β-D-ribofuranoside)	Sigma-Aldrich	D1916

**Critical commercial assays**		

RiboMax Large Scale RNA Production System-T7	Promega	P1320
Click-iT™ RNA Alexa Fluor™ 488 Imaging Kit	Thermo Fisher Scientific	C10329
Cytiva Amersham™ ECL™ Prime Western Blotting Detection Reagent	Cytiva	RPN2232

**Experimental models: Organisms/strains**		

Mouse/CB6F1(BALB/cA x C57B/6J)		N/A
Mouse/BALB/cA (wild type)	CLEA Japan	https://www.clea-japan.com/products/inbred/item_a0430
Mouse/C57B/6J (wild type)	CLEA Japan	https://www.clea-japan.com/products/inbred/item_a0420
Mouse/R26-H2B-EGFP (Accession No.CDB0238K)	RIKEN Center for Biosystems Dynamics Research	CDB0238K
Mouse/BALB/cA-R26-H2B-EGFP N4+	This paper	N/A
Mouse/C57BL/6J-R26-H2B-EGFP N4+	This paper	N/A

**Recombinant DNA**		

Plasmid: pcDNA3.1-pA for mRNA production	Dr. Kazuo Yamagata	N/A
Plasmid: mRFP1-tagged histone H2B	Dr. Hiroshi Kimura	N/A
Plasmid: EGFP-tagged α-tubulin	Dr. Kazuo Yamagata	N/A
Plasmid: EGFP-tagged Anillin-Cterminal	Dr. Ryota Uehara	N/A

**Software and algorithms**		

Fiji (ImageJ)	National Institutes of Health	https://imagej.nih.gov/ij/
Microsoft Excel	UTokyo Microsoft License	https://utelecon.adm.u-tokyo.ac.jp/microsoft/
GraphPad Prism 10	GraphPad Software	https://www.graphpad.com/
Adobe Illustrator	Adobe Creative Cloud	https://www.adobe.com/jp/creativecloud.html

**Others**		

Delta Vision Core System	Applied Precision	N/A
CoolSNAP HQ camera	Roper Scientific	N/A
Olympus IX71 microscope (20×/0.85 NA oil objective lens)	Olympus	N/A
Olympus IX70 microscope (20×/0.85 NA oil objective lens)	Olympus	N/A
Olympus IX70 microscope (60×/1.30 Sil oil objective lens)	Olympus	N/A
CO_2_ microscope stage incubator	TOKAI HIT	N/A
CCD camera iXon DU897E-CSO-#BV	ANDOR	N/A
MetaMorph	Universal Imaging	N/A
CSU10	Yokogawa	N/A
CSU-X1	Yokogawa	N/A
CellVoyager CV1000	Yokogawa	N/A
Piezo-driven micromanipulator	Prime Tech	N/A
LF101 electric cell fuser	BEX Co.	N/A
1-mm fusion chamber	BEX Co.	N/A
P-1000IVF	Sutter Instrument	N/A
Microforge MF-900	Narishige	N/A
ImageQuant LAS 4000 mini	GE HealthCare Japan	N/A

### Experimental model and study participant details

Unless otherwise noted, CB6F1 wild-type female mice (8–16 weeks old), generated by crossing female BALB/cA and male C57BL/6J mice, were used for oocyte collection. R26-H2B-EGFP mice, provided by the RIKEN Center for Biosystems Dynamics Research,^52^ were backcrossed with BALB/cA and C57BL/6J mice for more than four generations to generate R26-H2B-EGFP mice with a CB6F1 background. All mice were group-housed under standard conditions with a 12-hour light/dark cycle. All animal experiments were approved by the Animal Experimentation Committees of the Graduate School of Arts and Sciences (approval no. 26-29) and the Graduate School of Science (approval no. A2024S010), The University of Tokyo, and conducted in accordance with the guidelines for animal use issued by the Committee of Animal Experiments at The University of Tokyo. No cell lines or human participants were used in this study.

### Method details

#### Superovulation and collection of metaphase II oocytes

Female mice were superovulated by intraperitoneal injection of 5 IU PMSG, followed 48 hours later by 5 IU hCG. Cumulus–oocyte complexes were collected from the oviduct 16–20 hours after hCG injection. Cumulus cells were removed using M2 medium containing 100 μg/mL hyaluronidase. Denuded oocytes were washed and cultured in M16 medium at 37°C in 5% CO_2_. Unless otherwise stated, media were prepared as microdrops under paraffin oil in Petri dishes. Oocytes showing fragmentation, spontaneous activation, atrophy, or large perivitelline space were excluded.

#### Sr^2+^-induced parthenogenetic activation

Sr^2+^-induced parthenogenetic activation was performed using M16 medium supplemented with 5 mM SrCl_2_ and 5 mM EGTA.^53^ For Dip embryo generation, 2.5 μg/mL cytochalasin B (with 0.5% dimethyl sulfoxide, DMSO) was added to inhibit second polar body extrusion. For Halved-Hap embryos, Metaphase-II oocytes were preincubated in M16 with 250 μM CK-666 (with 0.5% DMSO) for 3 hours, then transferred to activation medium containing CK-666.^54^ To prevent CK-666 diffusion into paraffin oil, all CK-666 treatments were performed in 96-well plates without oil.

#### Electrofusion and electrical parthenogenetic activation

Fusion of Meta-II oocytes was performed following a protocol adapted from a previous study.^38^ Oocytes were treated with acid Tyrode’s solution to remove the zona pellucida, followed by incubation in M2 medium containing 50 μg/mL phytohemagglutinin for 1 minute. Paired oocytes were transferred to a 1-mm fusion chamber connected to the LF101 electric cell fuser. Fusion was induced using 5 V alternating current at 0.5 MHz for 5 seconds, followed by a 60 V direct current pulse for 50 μs. Using M2 medium in the chamber resulted in simultaneous fusion and parthenogenetic activation. After electrofusion, the oocytes were transferred to M16 medium with or without 2.5 μg/mL cytochalasin B, to generate Doubled-Tet or Doubled-Dip, respectively. For the fusion or stimulation of blastomeres in 2-cell-stage embryos, embryos were subjected to 5 V alternating current at 0.5 MHz for 5 seconds, followed by a 160 V direct current pulse for 40 μs in an M2 medium-filled fusion chamber. After electrofusion, the embryos were transferred to M16 medium for subsequent culture.

#### Enucleation

Embryos were placed in M2 medium containing 5 μg/mL cytochalasin B for approximately 10 minutes prior to enucleation. A glass needle (5–10 μm in diameter) was used to penetrate the plasma membrane under a micromanipulator and Piezo Impact Drive. The nucleus was aspirated and gently removed. To perform enucleation during the late G2 phase, embryos were transferred to M16 medium containing 20 μM CDK1 inhibitor Ro-3306 two hours before the average time of mitotic entry, as determined by live-cell imaging in this study. After enucleation, embryos were transferred to M16 medium containing 20 μM Ro-3306 for at least 1 hour to allow recovery from manipulation, followed by washing in M16 medium to remove the inhibitor before further culture. Sham operations for late G2 phase enucleation were conducted under identical conditions, including drug treatment and room temperature exposure. For late G2 phase embryos at the 1-cell stage, sham operations involved piercing the region between the two pronuclei 2–3 times using needles of the same diameter as in the experimental group. For 2-cell stage late G2 phase embryos, sham operations followed the same inhibitor treatment and room temperature exposure as the experimental group.

#### mRNA preparation and microinjection

mRNAs were synthesized *in vitro* from linearized template plasmids using the RiboMax™ Large Scale RNA Production System-T7, supplemented with Ribo m^7^G Cap Analog. Synthesized RNAs were purified by phenol extraction followed by ethanol precipitation.^55^ The template plasmids for EGFP-tagged α-tubulin or mRFP1-tagged histone H2B were provided from Kazuo Yamagata.^56^ The template plasmid for EGFP-Anillin-Cterminal was constructed using cDNA from a pEGFP-Anillin-Cterminal vector^57^ and pcDNA3.1-polyA vector.^56^^,^^58^ Few picoliters of mRNAs (10–200 ng/μl) were injected into Meta-II oocytes using a Piezo-driven micromanipulator with glass needles approximately 3 μm in diameter.

#### Live imaging

Confocal imaging was performed using an 20× lens equipped with a spinning disk confocal system (CSU-10 or CSU-X1) and a CO_2_ microscope stage incubator. Images were captured with a CCD camera controlled by MetaMorph software. In imaging experiments involving EGFP–α-tubulin, some observations were conducted using the CellVoyager CV1000 confocal scanner box. Time-lapse imaging included a single mid-plane bright-field reference and one or two fluorescence channels acquired at 4-8 μm intervals (11-21 Z-sections). Time intervals ranged from 2.5 minutes to 15 minutes. As 1-cell stage embryos were highly light-sensitive and often failed to develop beyond the 2-cell stage, imaging of the first and subsequent mitoses was performed separately.

#### Click-iT EU RNA labeling assay

Nascent RNA labeling was performed using the Click-iT RNA Alexa Fluor 488 Imaging Kit according to the manufacturer’s instructions, with minor modifications. Embryos were incubated in M16 medium supplemented with 1 mM EU for 1 h or 0.5 mM EU for 10 h. After incubation, embryos were fixed and subjected to click chemistry detection following the kit protocol.

Z-stack images of 2-cell embryos were acquired under identical imaging settings. To ensure consistency, embryos were oriented laterally whenever possible, so that the two blastomeres were oriented side by side. Z-projections were generated using the Sum slices option in Fiji, and the integrated density of EU signals was measured at the center of each nucleus. Nuclear boundaries were defined using Hoechst staining. Corrected integrated density (Corrected IntDen) was calculated as: Corrected IntDen = RawIntDen (sample) – RawIntDen (negative control). The negative control was derived from embryos in the same staining batch processed without EU incorporation. Corrected integrated density values of individual nuclei were plotted as independent data points.

#### Transcription-inhibitor treatment

2-cell stage diploid embryos were treated with α-amanitin (1, 5, 10, or 25 μg/mL) or DRB (1.6, 8, 10, 20, or 40 μM with 0.5% DMSO) in M16 medium from 20 h post-activation (hpa) until 44–48 hpa.

#### Immunofluorescence

After removal of the zona pellucida using acidic Tyrode’s solution, oocytes or embryos were washed with 0.5% polyvinyl pyrrolidone in PBS and subsequently fixed in 4% paraformaldehyde for 30–40 minutes at 25°C. Fixed samples were permeabilized with 0.25% Triton X-100 in PBS for 10–15 minutes at 25°C, followed by blocking in PBS containing 3% BSA and 0.1% Tween-20 for 1 hour at 25°C or overnight at 4°C. For BrdU staining, cells were treated with 2 M HCl and 0.5% Triton X-100 in PBS for 45 minutes.

After blocking, samples were incubated with primary antibodies, including anti-BrdU (1:250), anti-α-tubulin (YOL1/34, 1:1000), anti–Lamin B1 (B-10, Alexa Fluor 488, 1:500), and anti–phospho-Histone H2A.X (Ser139, 1:1000). After washing, samples were incubated with appropriate fluorescently labeled secondary antibodies (1:1000), together with Hoechst 33342 (5 μg/mL) or rhodamine–phalloidin (1:1000) for 1–2 h at 25 °C.

Each incubation step was followed by three 5-min washes in PBS containing 0.1% BSA and 0.1% Tween-20. Samples were observed at 25°C using a fluorescence microscope equipped with 60× or 20× oil immersion objective lenses and a CoolSNAP HQ camera. The system was controlled by DeltaVision SoftWorx software. Fluorescence images were acquired as Z-stacks at 1–2 μm intervals and displayed as maximum intensity Z-projections. The imaging medium consisted of PBS supplemented with 0.1% BSA.

#### Western blotting

Ten or twenty 30-hpa haploid or diploid embryos were lysed in Laemmli sample buffer and subjected to SDS–PAGE on 10% gels. Proteins were transferred semidry onto PVDF membranes using transfer buffer containing 20% methanol. Membranes were blocked with 5% non-fat dry milk in Tris Buffered Saline with Tween 20 (TBS-T) and incubated with primary antibodies in TBS-T: anti-TUBA4A (1:5000) and anti-β-actin (1:1000). After TBS-T washes, membranes were incubated with HRP-conjugated sheep anti-mouse IgG (1:4000). Signals were developed using ECL Prime detection reagent and visualized with ImageQuant LAS 4000 mini.

For quantification, each data point represents the intensity ratio of paired Dip and Hap bands located adjacent to each other on the same PVDF membrane, thereby minimizing variation caused by experimental differences or electrophoretic position.

#### Image analysis

Image analysis was performed using Fiji. The volumes of blastomeres and nuclei were quantified by manually selecting their largest cross-sectional areas, estimating the area, and approximating the structures as spheres. The timing of nuclear envelope breakdown (NEBD) and anaphase onset, as well as the presence of chromosome segregation errors, were determined from fluorescence images of histone H2B. The success of cytokinesis was assessed using bright-field images. Blastomeres that did not enter mitosis were excluded from cell cycle-related analyses. For blastomeres exhibiting severe chromosome segregation failure, anaphase onset was defined as the first frame showing evident chromosome displacement. The timing of contractile ring formation was calculated as the interval between the initial accumulation of EGFP–anillin at the cortex and its constriction into a single point.

In live imaging, bright-field images were captured from a single slice near the middle of each sample and are presented as references to facilitate visualization of blastomere boundaries. Unless otherwise specified, fluorescence images represent Z-projections of the entire sample. For merged still images from live-cell imaging, the contrast of the bright-field channel was substantially reduced to better visualize the fluorescent signals. Unprocessed data can be found in the corresponding videos.

For some immunofluorescence and live-cell images, contrast was adjusted uniformly across the entire image to enhance visualization, without any local modifications. Signals of the target structures were neither eliminated nor saturated by these adjustments.

### Quantification and statistical analysis

Data are presented as mean ± SD (or mean with Wilson/Brown 95% CI where indicated). Statistical analyses were performed using GraphPad Prism 10. Pairwise comparisons of proportions were assessed using two-sided Fisher’s exact tests. Comparisons of continuous variables were performed using unpaired two-tailed t-tests, Mann–Whitney U tests, or Kruskal–Wallis tests followed by Dunn’s multiple comparisons tests, as appropriate. Statistical significance is denoted as follows: p < 0.05 (∗), p < 0.01 (∗∗), p < 0.001 (∗∗∗), p < 0.0001 (∗∗∗∗); n.s., not significant. Sample sizes are indicated in parentheses above each bar or within the plot.
