# Supplementary material for: Balanced DNA-to-cytoplasm ratio at the 2-cell stage is critical for mouse preimplantation development
Source: iScience. 2025 Dec 12;29(1):114416. doi: 10.1016/j.isci.2025.114416 (PMC12800424; doi:10.1016/j.isci.2025.114416)
Supplement: Document S1. Figures S1–S6 [file mmc1.pdf]

iScience, Volume *29*

Supplemental information

Balanced DNA-to-cytoplasm ratio at the 2-cell stage is critical for mouse preimplantation development

Tao Pan (潘韬), Natsumi Taira (平良夏実), and Miho Ohsugi (大杉美穂)

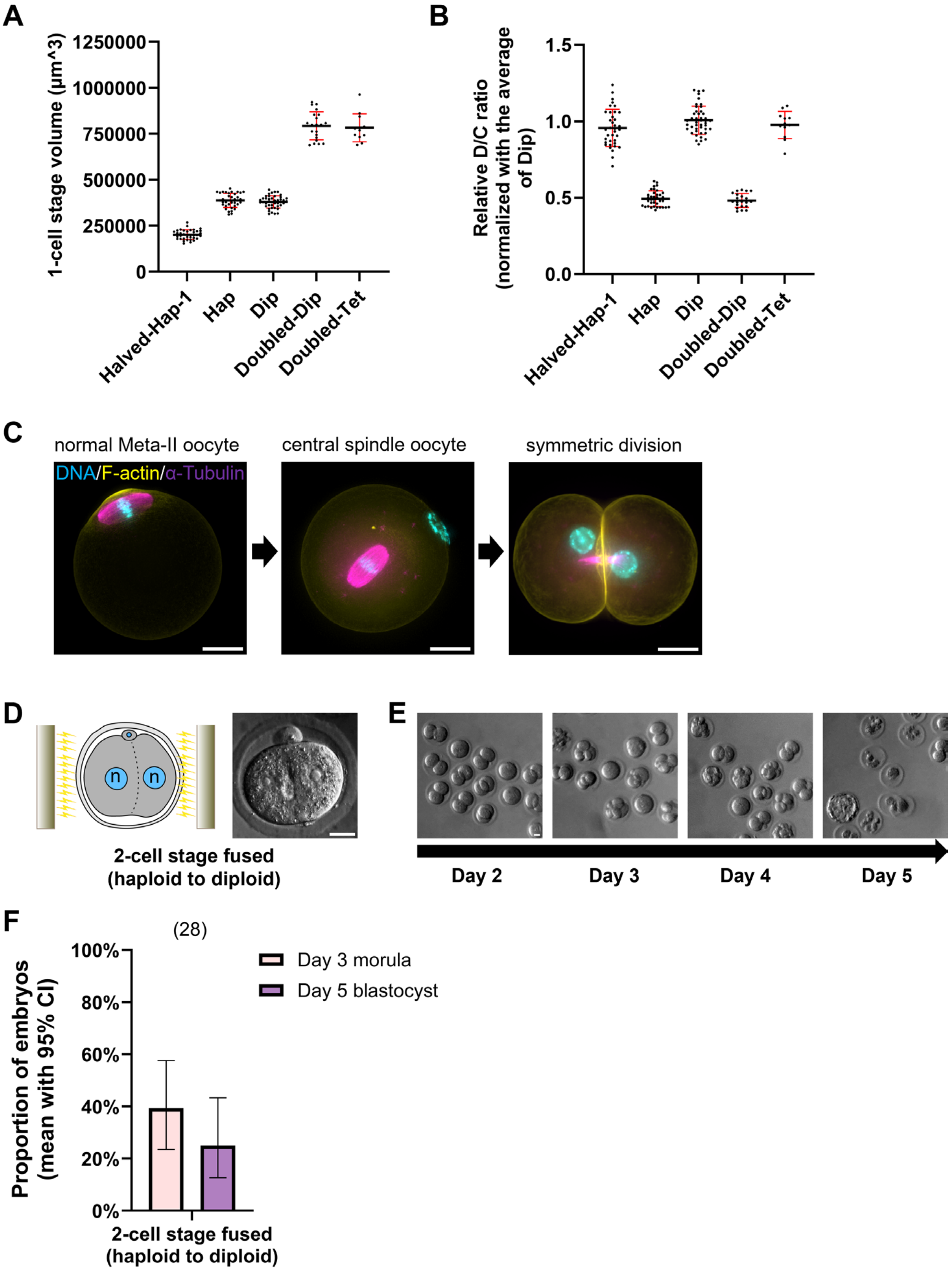
A
1-cell stage volume (µm^3)
1250000
1000000
750000
500000
250000
0
Halved-Hap-1
Hap
Dip
Doubled-Dip
Doubled-Tet
B
Relative D/C ratio
(normalized with the average
of Dip)
1.5
1.0
0.5
0.0
Halved-Hap-1
Hap
Dip
Doubled-Dip
Doubled-Tet
C
normal Meta-II oocyte
central spindle oocyte
symmetric division
DNA/F-actin/α-Tubulin
D
n
n
2-cell stage fused
(haploid to diploid)
E
Day 2
Day 3
Day 4
Day 5
F
(28)
Day 3 morula
Day 5 blastocyst
Proportion of embryos
(mean with 95% CI)
100%
80%
60%
40%
20%
0%
2-cell stage fused
(haploid to diploid)

Figure S1. Validation of D/C ratio manipulation and evaluation of developmental outcomes in 2-cell-stage fused haploid embryos. Relates to Figure 1

(A and B) The volume of the embryos was calculated by manually outlining 1-cell stage embryos that appeared approximately circular and assuming them to be spheres. The D/C ratio was calculated by setting the ratio of the average volume of Dip to its ploidy as 1.0 (mean ± SD, n ≥ 12; ≥ 3 experiments).

(C) Representative immunofluorescence images of Halved-Hap production. DNA is shown as cyan, α-tubulin is shown as magenta, and F-actin is shown as yellow. For the central spindle oocyte (middle), an image stack was acquired at approximately 30 μm from the middle part of the sample.

(D and E) Schematic representation and representative bright-field images of 2-cell stage electrofused diploid embryo.

(F) Day 3 morula rate and Day 5 blastocyst rate of 2-cell stage S phase electrofused diploid embryo (mean with Wilson/Brown 95% CI; 2 experiments).

Scale bars, 20 μm. Sample sizes are shown in parentheses above each bar.

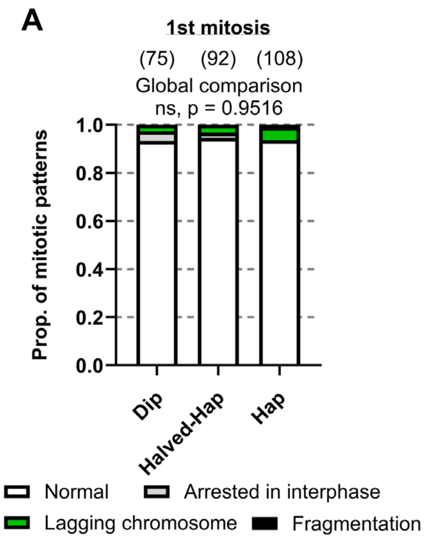

A
1st mitosis
(75) (92) (108)
Global comparison
ns, p = 0.9516
Prop. of mitotic patterns
1.0
0.8
0.6
0.4
0.2
0.0
Dip
Halved-Hap
Hap
Normal
Arrested in interphase
Lagging chromosome
Fragmentation
B
**
ns
****
Time from activation to the second mitosis NEBD (h)
50
45
40
35
30
Dip
Halved-Hap
Hap
C
****
ns
***
Time from the second mitosis NEBD to anaphase (min)
300
200
100
0
Dip
Halved-Hap
Hap

Figure S2. Haploid embryos did not exhibit abnormalities during the first mitosis and showed prolonged interphase and mitosis at the 2-cell stage. Relates to Figure 2.

(A) Proportion of mitotic patterns obtained by time-lapse imaging (mean; 3 experiments). Global differences of proportion of normal blastomeres across groups were evaluated with the Fisher–Freeman–Halton exact test.

(B and C) Scatterplots showing the time from activation to NEBD of the second mitosis, and the time from NEBD to anaphase during the second mitosis shown in Fig. 2D and E (mean ± SD). Statistical analysis was performed using the Kruskal-Wallis test to confirm overall significant differences among groups, followed by pairwise comparisons using Dunn's multiple comparisons test to account for multiple comparisons.

Statistical significance is denoted as follows: $p < 0.01$ (**), $p < 0.001$ (***), $p < 0.0001$ (****); n.s., not significant.

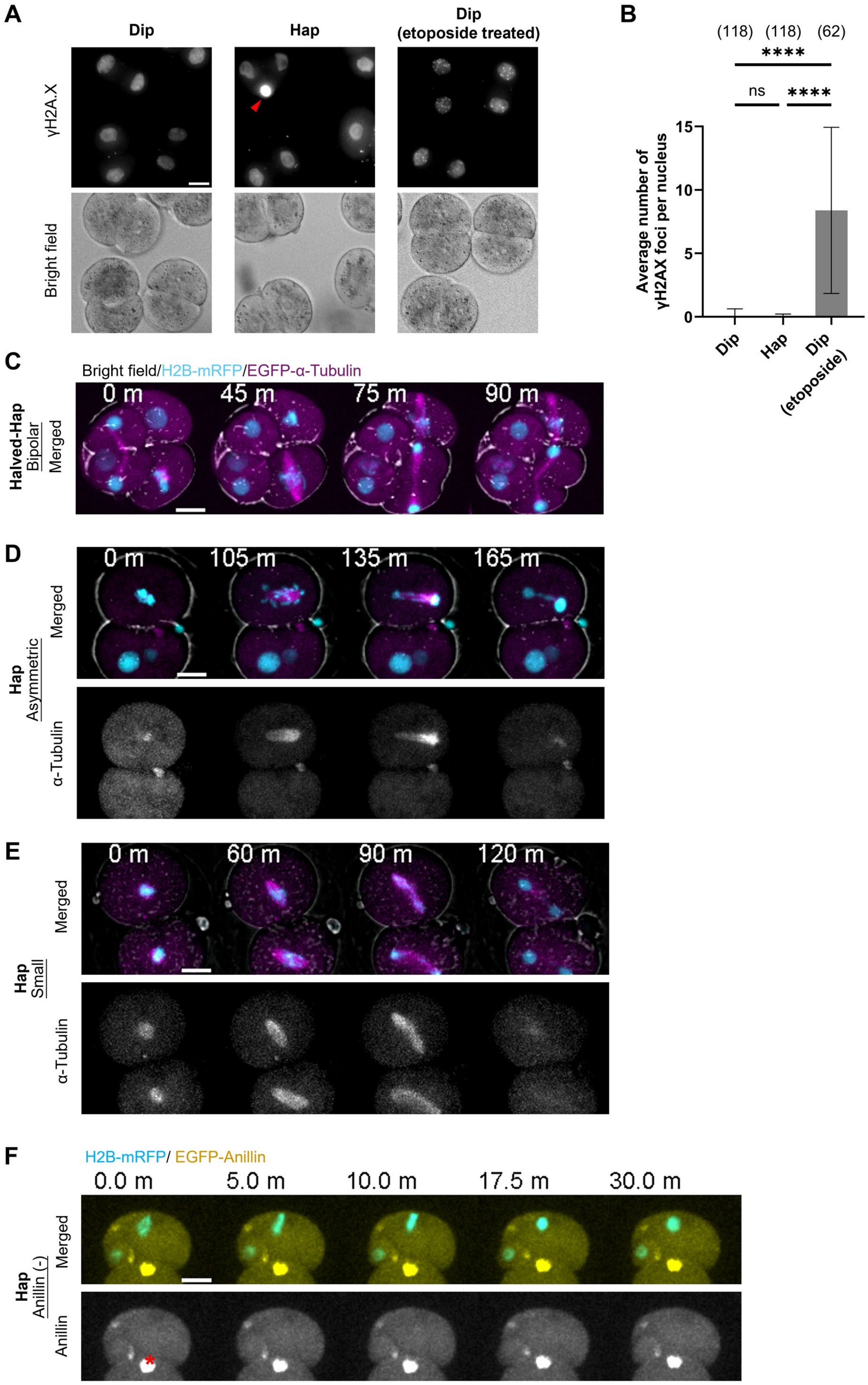

A
Dip
Hap
Dip
(etoposide treated)
γH2A.X
Bright field
B
(118) (118) (62)
****
ns
****
Average number of
γH2AX foci per nucleus
15
10
5
0
Dip
Hap
Dip
(etoposide)
C
Bright field/H2B-mRFP/EGFP-α-Tubulin
Halved-Hap
Bipolar
Merged
0 m
45 m
75 m
90 m
D
0 m
105 m
135 m
165 m
Hap
Asymmetric
Merged
α-Tubulin
E
0 m
60 m
90 m
120 m
Hap
Small
Merged
α-Tubulin
F
H2B-mRFP/ EGFP-Anillin
0.0 m
5.0 m
10.0 m
17.5 m
30.0 m
Hap
Anillin (-)
Merged
Anillin

Figure S3. Haploid embryos exhibit spindle and contractile ring associated abnormalities during the second mitosis without detectable DNA damage. Relates to Figure 3

(A) Representative immunofluorescence images showing γH2AX signals in Dip, Hap, and etoposide-treated Dip. For the etoposide-treated Dip group, embryos were exposed to 5 μM etoposide from 24 to 25 hpa. All samples were fixed at 30 hpa. Red arrow indicates the second polar body in Hap.

(B) Quantification of γH2AX foci in the samples mentioned in C (mean ± SD, 2 experiments). Statistical analysis was performed using the Kruskal-Wallis test to confirm overall significant differences among groups, followed by pairwise comparisons using Dunn's multiple comparisons test to account for multiple comparisons. Statistical significance is denoted as follows: $p < 0.0001$ (****); n.s., not significant.

(C-E) Still images from time-lapse imaging of the second mitosis in microinjected embryos expressing histone H2B-mRFP1 and EGFP–α-tubulin. Timestamps indicate time (minutes) since NEBD. See also Video S2. Time-lapse imaging of this part was captured using the confocal scanner unit CSU10, which has a slightly different resolution compared to the other imaging data obtained with the confocal scanner unit CSU-X1 in this study.

(F) Still images from time-lapse imaging of the second mitosis in microinjected embryos expressing histone H2B-mRFP1 and EGFP-Anillin. Red asterisk, aggregates of EGFP–Anillin signals present on the cell membrane from interphase. Timestamps indicate time (minutes) since anaphase. See also Video S3.

Scale bars = 20 μm, sample sizes are indicated in parentheses above each bar.

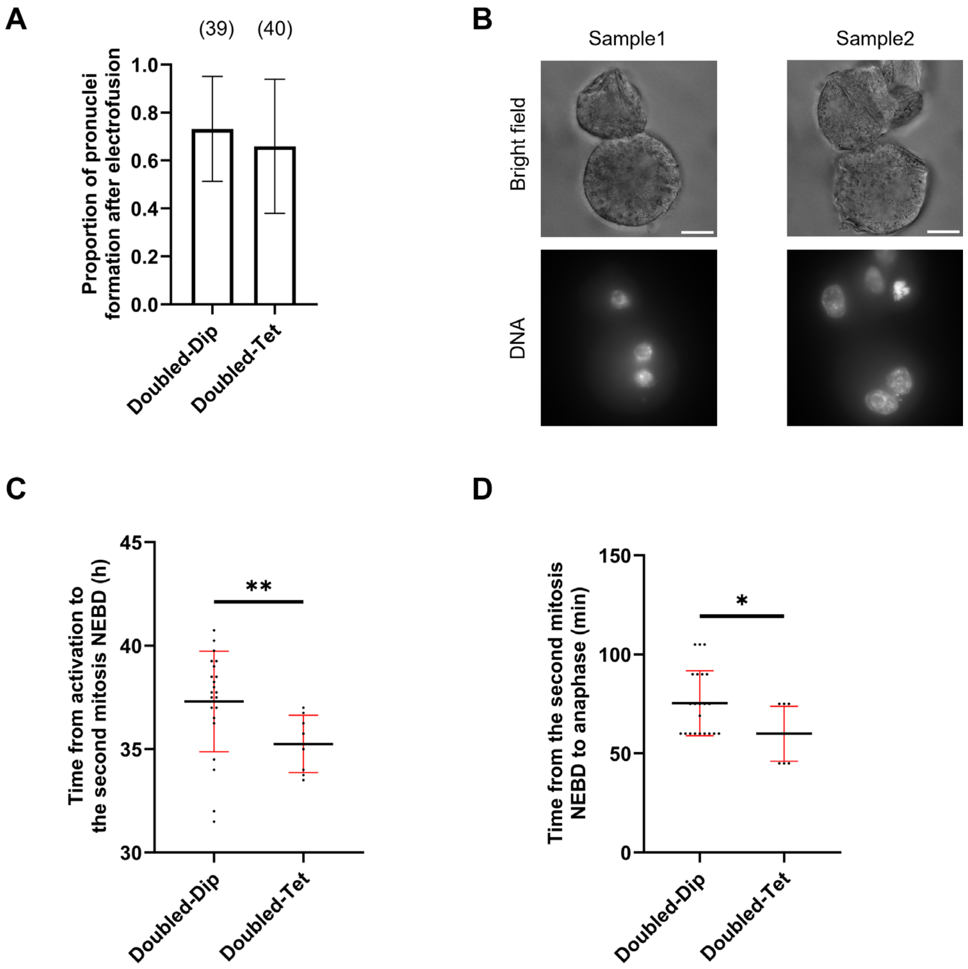
A
(39)
(40)
Proportion of pronuclei formation after electrofusion
1.0
0.8
0.6
0.4
0.2
0.0
Doubled-Dip
Doubled-Tet
B
Sample1
Sample2
Bright field
DNA
C
Time from activation to the second mitosis NEBD (h)
45
40
35
30
**
Doubled-Dip
Doubled-Tet
D
Time from the second mitosis NEBD to anaphase (min)
150
100
50
0
*
Doubled-Dip
Doubled-Tet

Figure S4. Electrofused diploid embryos exhibit abnormal nuclear morphology and delayed mitosis compared to tetraploid counterparts. Relates to Figure 4

(A) Average proportion of pronuclei formation in electrofused embryos (mean ± SD, ≥ 6 experiments).

(B) Two fixed samples from arrested Doubled-Dip. DNA was stained with Hoechst. Since Doubled-Dip samples are scarce, only those that were confirmed to be unable to develop beyond the morula stage were selected for fixation. As a result, there may be a 48-hour interval between the occurrence of developmental arrest and fixation. Scale bars = 20 μm.

(C and D) Scatterplots showing the time from activation to NEBD of the second mitosis, and the time from NEBD to anaphase during the second mitosis of double-sized embryos shown in Fig. 4E and F (mean ± SD). Statistical analysis was performed using the Mann-Whitney U test to compare the two groups, $p < 0.05$ (*), $p < 0.01$ (**).

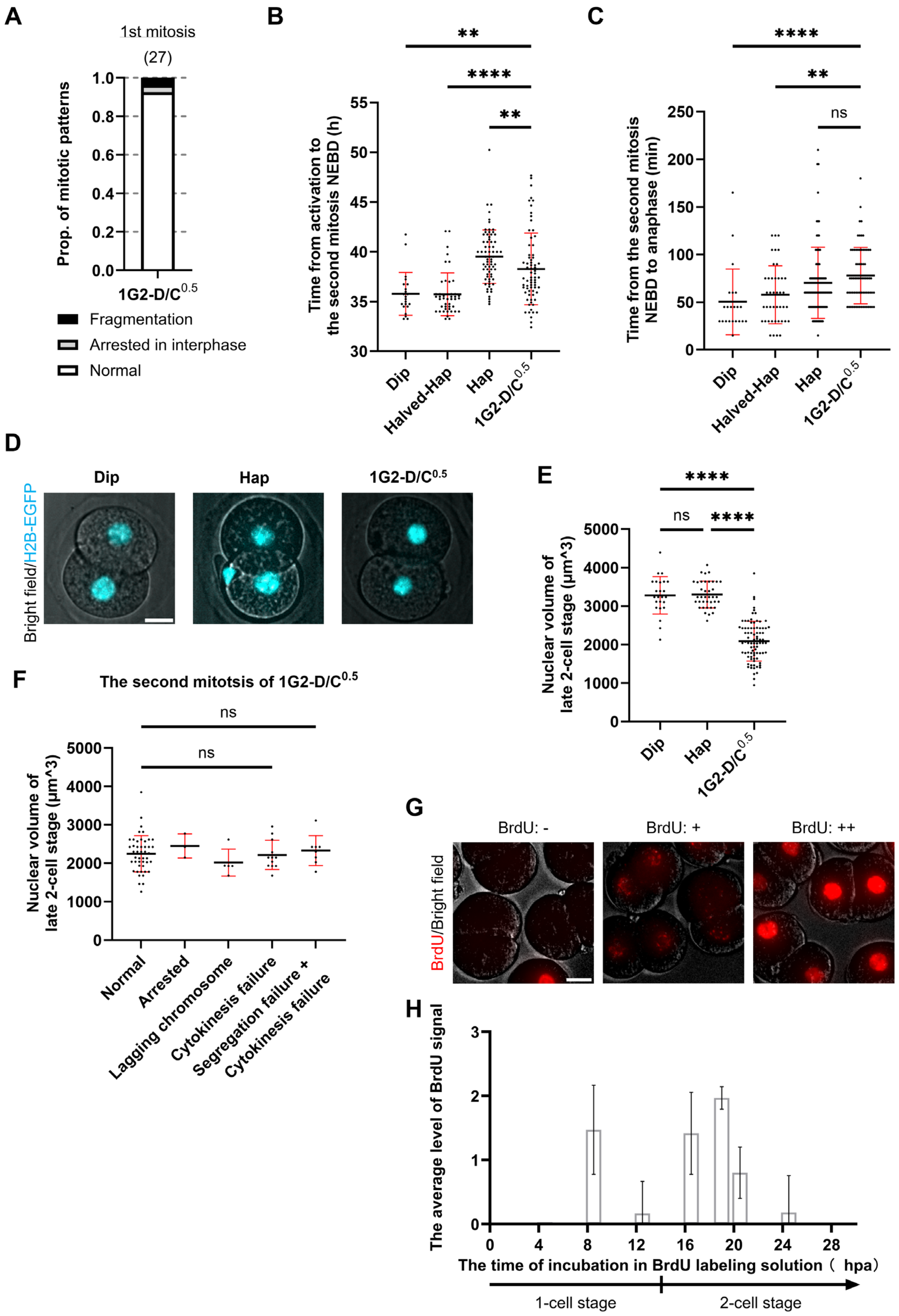
A
1st mitosis
(27)
Prop. of mitotic patterns
1G2-D/C0.5
Fragmentation
Arrested in interphase
Normal
B
Time from activation to the second mitosis NEBD (h)
Dip
Halved-Hap
Hap
1G2-D/C0.5
C
Time from the second mitosis NEBD to anaphase (min)
ns
D
Dip
Hap
1G2-D/C0.5
Bright field/H2B-EGFP
E
Nuclear volume of late 2-cell stage (μm^3)
ns
F
The second mitotsis of 1G2-D/C0.5
Normal
Arrested
Lagging chromosome
Cytokinesis failure
Segregation failure + Cytokinesis failure
G
BrdU: -
BrdU: +
BrdU: ++
BrdU/Bright field
H
The average level of BrdU signal
The time of incubation in BrdU labeling solution（ hpa）
1-cell stage
2-cell stage

Figure S5. Embryos with a halved D/C ratio at the G2 phase of the 1-cell stage exhibit a prolonged 2-cell stage and reduced nuclear size compared to diploids. Relates to Figure 5 and 6.

(A) Proportion of mitotic patterns obtained by time-lapse imaging (mean; 3 experiments). See related Video S5.

(B and C) Scatterplots showing the time from activation to NEBD of the second mitosis, and the time from NEBD to anaphase during the second mitosis in 1G2-D/$C^{0.5}$ (mean ± SD). The data used for comparison with the 1G2-D/$C^{0.5}$ group is identical to Fig, S2B and C. Statistical analysis was performed using the Kruskal-Wallis test to confirm overall significant differences among groups, followed by pairwise comparisons using Dunn's multiple comparisons test to account for multiple comparisons.

(D) Still images from time-lapse imaging at the late G2 phase of the 2-cell stage in three types of embryo models. Embryos expressing histone H2B-EGFP.

(E) Scatterplots showing the nuclear volume of three embryo models at the late G2 phase of the 2-cell stage (mean ± SD). Nuclear volume was calculated by manually outlining nuclei that appeared approximately circular and assuming them to be spheres. Statistical analysis was performed using the One-way ANOVA to confirm overall significant differences among groups, followed by pairwise comparisons using Dunn's multiple comparisons test to account for multiple comparisons.

(F) Scatterplots showing the nuclear volume of 1G2-D/$C^{0.5}$ (mean ± SD). The data are the same as those presented in E but classified based on the status of the second mitosis. Statistical analysis was performed using the 2-way ANOVA to confirm overall significant differences among groups, followed by pairwise comparisons using Dunn's multiple comparisons test to account for multiple comparisons.

(G) Immunofluorescence images of BrdU signals in Dip embryos incubated for 1 h with 0.2 mM BrdU at different time points.

(H) Quantification of BrdU signal levels (mean with SD; $n \geq 10$ per time point; 1 experiment). Nuclei exhibiting uniform fluorescence signals were scored as "++" (2 points), those with partial nuclear signals as "+" (1 point), and those without detectable signals as "–" (0 points).

Scale bars = 20 µm, sample sizes are indicated in parentheses above each bar.

Statistical significance is denoted as follows: $p < 0.01$ (**), $p < 0.0001$ (****); n.s., not significant.

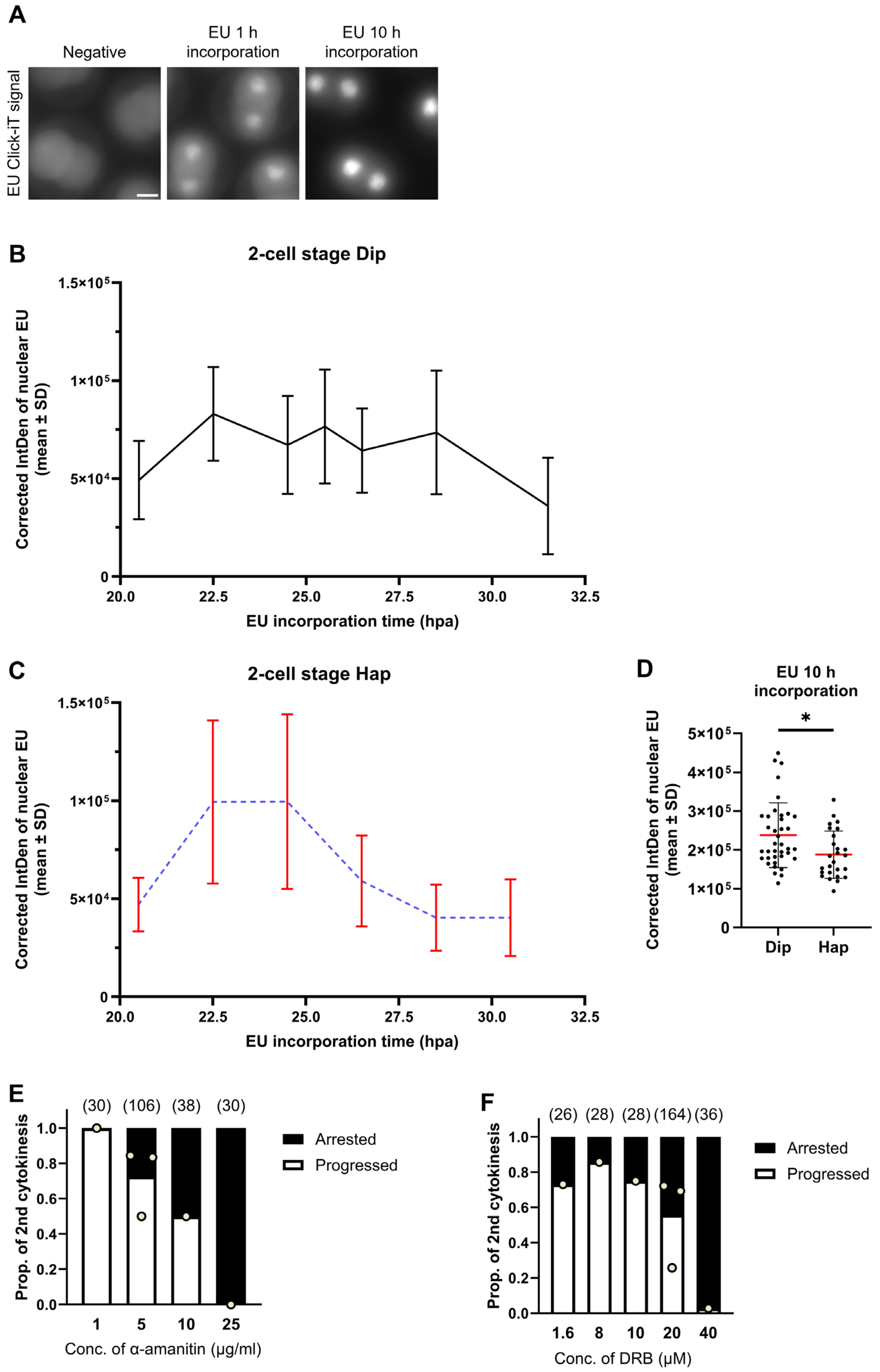
A
Negative
EU 1 h incorporation
EU 10 h incorporation
EU Click-iT signal
B
2-cell stage Dip
Corrected IntDen of nuclear EU (mean ± SD)
1.5×10⁵
1×10⁵
5×10⁴
0
20.0
22.5
25.0
27.5
30.0
32.5
EU incorporation time (hpa)
C
2-cell stage Hap
Corrected IntDen of nuclear EU (mean ± SD)
EU incorporation time (hpa)
D
EU 10 h incorporation
*
Corrected IntDen of nuclear EU (mean ± SD)
5×10⁵
4×10⁵
3×10⁵
2×10⁵
1×10⁵
0
Dip
Hap
E
(30) (106) (38) (30)
Prop. of 2nd cytokinesis
1.0
0.8
0.6
0.4
0.2
0.0
1
5
10
25
Conc. of α-amanitin (μg/ml)
Arrested
Progressed
F
(26) (28) (28) (164) (36)
Prop. of 2nd cytokinesis
1.6
8
10
20
40
Conc. of DRB (μM)
Arrested
Progressed

Figure S6. Haploid embryos at the 2-cell stage exhibit a shorter transcriptional peak duration and lower transcriptional output compared to diploids. Relates to Figure 7.

(A) Z-projections of EU-Click-iT immunofluorescence signals were generated using Sum Slices. Scale bar = 20 μm.

(B and C) Quantification of corrected integrated densities of embryos incubated for 1 h with EU at different time points (mean ± SD; $n \geq 16$ per time point; 1 experiment)

(D) Quantification of corrected integrated densities of embryos incubated for 10 h with EU (mean ± SD; $n \geq 26$; 1 experiment). Statistical analysis was performed using the Mann–Whitney test to compare Hap and Dip groups (two-tailed), $p < 0.05$ (*).

(E and F) Proportion of cytokinesis during the second mitosis in embryos treated with different concentrations of transcriptional inhibitors (mean; each dot represents the proportion of progressed blastomeres from one experiment). Sample sizes are indicated in parentheses above each bar.
